# Symptomatological Variants and Related Clinical Features in Adult Attention Deficit Hyperactive Disorder

**DOI:** 10.3390/ijerph18030922

**Published:** 2021-01-21

**Authors:** Alessandro Pallucchini, Marco Carli, Marco Scarselli, Icro Maremmani, Giulio Perugi

**Affiliations:** 1 PISA-School of Experimental and Clinical Psychiatry, 56100 Pisa, Italy; pallucchini.a@gmail.com; 2 Department of Clinical and Experimental Medicine, School of Clinical Pharmacology, University of Pisa, 56100 Pisa, Italy; carlimarco@outlook.it; 3 Department of Translational Research and New Technologies in Medicine and Surgery, University of Pisa, 56100 Pisa, Italy; marco.scarselli@med.unipi.it; 4 Association for the Application of Neuroscientific Knowledge to Social Aims (AU-CNS), 55045 Pietrasanta, Lucca, Italy; 5 Vincent P. Dole Dual Disorder Unit, 2nd Psychiatric Unit, Santa Chiara University Hospital, University of Pisa, 56100 Pisa, Italy; 6 G. De Lisio Institute of Behavioral Sciences, 56100 Pisa, Italy; 7 Saint Camillus International University of Health and Medical Sciences—UniCamillus, 00131 Rome, Italy; 8 2nd Psychiatric Unit, Department of Clinical and Experimental Medicine, Santa Chiara University Hospital, University of Pisa, 56100 Pisa, Italy; giulio.perugi@med.unipi.it

**Keywords:** attention deficit/hyperactivity disorder, emotional dysregulation, substance user, positive emotionality

## Abstract

A large amount of the current literature has focused on the characteristic symptoms of attention deficit hyperactivity disorder (ADHD) in children and adolescents. In contrast, less attention has been devoted to ADHD clinical subtypes in adult patients. We evaluated 164 consecutive adult ADHD (A-ADHD) outpatients using DSM-5 criteria and many specific rating scales and questionnaires. A principal component factor analysis was performed on clinical and symptomatological variables to describe potential clinical variants. We sought to determine different A-ADHD variants focusing on demographic and clinical features. A four-factor solution was identified, and patients were clustered, according to their z-score, in 4 subgroups. The first was marked out by Emotional Dysregulation (ED), the second by Substance Use (SU), the third by Core-ADHD Symptoms (Co-ADHD) and the fourth by Positive Emotionality (PE). Predominantly ED patients showed worse overall function, early treatment with antidepressants and a greater presence of borderline personality disorder than predominantly Co-ADHD patients. Predominantly SU patients reported high rates of bipolar disorder and severe general psychopathology. The PE factor was related to hyperthymic temperament and hypomania and showed a higher level of functioning. Females with A-ADHD showed a lower risk of being included in SU, and A-ADHD patients with co-occurring delayed sleep phase had less risk of being included in the SU factor than the prevailing Co-ADHD group. Our empirically based description of four clinical A-ADHD variants shows several aspects beyond the definition given by the DSM-5 diagnostic criteria.

## 1. Introduction

Neurodevelopmental disorders include a broad group of disabilities involving some forms of disruption of brain development, and group together a very heterogeneous range of neuropsychiatric conditions where each condition may differ substantially from the others in clinical presentation and etiology [1]. Attention deficit hyperactivity disorder (ADHD) is the most common neurodevelopmental disorder and is characterized in children and adolescents by high levels of inattentiveness, impulsivity and/or hyperactivity.

The evolutionary trajectory of the disorder into adulthood is variable, and about half of children and young adolescents with ADHD show an attenuation of hyperactivity when they grow up [2]. Usually, patients with adult-ADHD (A-ADHD) are predominantly inattentive and impulsive [3]. In 15% of these cases, the full syndrome persists into adulthood [4].

In epidemiological surveys, the prevalence of A-ADHD is estimated to be around 2.5–4% [5,6] and the hyperactive/impulsive presentation is more common in males than in females [7]. A-ADHD patients come to clinical attention with a variety of symptoms of internal (anxiety, depression and social withdrawal) and external (problems in the self-control of emotions and behaviors such as defiant behavior, aggressiveness and disruptive impulses) psychiatric comorbidities [8,9], which form a severe and complex clinical picture [10], making their recognition and management a complicated task [11].

In the DSM-5, ADHD is described according to a two-dimensional model comprising inattentive, hyperactive-impulsive or combined subtypes. This model underestimates several clinical aspects of fundamental importance to the clinical management of adults with the disorder.

However, given the proposals made in scientific research, the current categorical approach to nosography may be intended to be overcome by a more appropriate etiopathogenetic classification system of mental diseases consistent with the existence of definite neurobiological processes that support a variety of psychiatric conditions that share the same pathophysiology. These pathophysiological traits may be explained by the clinic as transdiagnostic psychological/psychiatric defects and vulnerabilities that play the role of “endophenotypes”. In particular, The Research Domain Criteria (RDoC) project [12,13] developed by the National Institute of Mental Health (NIMH) has been made available to researchers in order to seek new ways to classify mental disorders by basing them on the transdiagnostic forms, and subconstructs that link neurobiological systems with their subunits (genes, molecules, neurons and neural circuits), to distinct but correlated functional and dysfunctional aspects.

Attractively, the RDoC provides a dimensional viewpoint on psychiatry, ranging from normal to pathological, including diagnostic subthreshold psychological/psychiatric settings, psychological/psychiatric precursors and predisposing factors. Prime patterns of these neurobiologically based systems considered in the RDoC transdiagnostic approach to the pathogenesis of addiction, and in mood and impulse control dysfunctions, are reward-processing systems and inhibitory systems.

Different variants of A-ADHD have been described, with different courses [14,15]. However, the presence of emotional dysregulation appears to be a significant component [16,17,18] that leads over time to various psychiatric comorbidities such as substance use disorders (SUD), mood disorders and personality disorders [19,20], and thus to greater severity in overall functioning [21,22,23].

The term “emotional dysregulation” is used to identify a series of affective and behavioral phenomena found in many psychiatric disorders [24]; it is often associated with a substantial syndromic complexity not mentioned in the DSM-5 criteria. The main features of emotional dysregulation are: irritability, with frequent short-term behavioral outbursts (temperamental control); short and unpredictable mood swings (affective instability); and reduced ability to manage stress with the feeling of being overwhelmed by emotions (emotional over-reactivity) [25].

The aim of this study is to explore the presence of different clinical patterns in a population of A-ADHD subjects so as to more accurately describe the heterogeneity of the disorder in adulthood.

## 2. Materials and Methods

### 2.1. Sample

This is a multicentric, cross-sectional, observational study. The sample included 164 consecutive outpatients admitted, with the same protocol, to Psychiatric Unit 2 at the University of Pisa or to the Department of Neurology and Psychiatry of the Sapienza University in Rome. All patients were diagnosed with A-ADHD according to DSM-5 criteria. No exclusion criteria were applied, except for the patient’s age (≤18 years old). Demographic data, psychiatric history, past psychiatric treatment, past ADHD specific treatment and self-reported drug use were recorded at study entry. Resident and senior psychiatrists of the ADHD research groups evaluated the recruited patients by holding a clinical interview and specific diagnostic tests. The study was conducted between 2016 and 2019, in accordance with the WMA Declaration of Helsinki—Ethical Principles for Medical Research Involving Human Subjects. All patients enrolled in the study filled in and signed an informed consent document. Both the consent form and the experimental procedures were approved by the ethics committee of the University of Pisa, in accordance with internationally accepted criteria for ethical research (study ID: 14003; code: ADHD-MOOD).

### 2.2. Instruments

The diagnosis of ADHD was made according to DSM-5 criteria and confirmed by using the Italian version of the semi-structured, clinician-administered Diagnostic Interview for ADHD in adults (DIVA 2.0). When possible, a caregiver/family member of the patient participated in the interview to provide retrospective and collateral information. Each interview comprised three parts: nine criteria for attention deficit, nine criteria for hyperactivity-impulsivity and a third part evaluating the age of onset and the level of impairment. The presence/absence of each item was investigated both in childhood/adolescence and adulthood. If collateral information provided by the caregiver was not available, the diagnosis was based exclusively on the patient’s recollection.

The clinical evaluation was based on the following scales:

- Conner’s Adult ADHD Rating Scales—Observer: Screening Version (CAARS-O:SV): this is a 30-item questionnaire for the caregiver/family member. It evaluates the presence of symptoms and behaviors associated with ADHD in adults, and rates symptoms on a Likert scale, ranging from 0 “never” to 3 “very often”. CAARS-O:SV is a tool designed for the DSM-V criteria, although there is no updated version yet. Nevertheless, it is appropriate to assess the severity of A-ADHD regardless of the version of the DSM. The Screening Version provides four subscale scores: Inattentive Symptoms, Hyperactive-Impulsive Symptoms, Total ADHD Symptoms and ADHD Index. Higher scores indicate clinically significant levels of ADHD symptoms [26].
- Adult ADHD Self-Report Scale (ASRS): this 18-question scale was used to assess the frequency of self-reported adult ADHD symptoms derived from the DSM-IV criteria. Answers are expressed on a Likert scale, ranging from 0 “never” to 4 “very often”, and a higher total score indicates significant levels of ADHD symptoms [27].
- Brief Temperament Evaluation of Memphis, Pisa, Paris and San Diego (brief TEMPS-M): this is the short version of the 110-item questionnaire evaluating the self-reported affective and anxious temperaments. The brief TEMPS-M consists of 35 items, with a five-point Likert scale ranging from 1 “not at all” to 5 “very much”. The questionnaire assesses five types of temperament: depressive, cyclothymic, hyperthymic, irritable and anxious temperaments [28].
- Barratt Impulsiveness Scale (BIS-11): this is a self-report questionnaire designed to investigate the personality/behavioral construct of impulsiveness and is considered the gold standard measure of impulse control. The BIS-11 consists of 30 questions; answers are expressed as a range going from 1 “never/rarely” to 4 “almost always/always”. The scale is structured following a factorial analysis covering six first-order (attention, cognitive instability, motor, perseverance, self-control, cognitive complexity) and three second-order factors (attentional, motor, non-planning impulsivity) [29].
- Difficulties in Emotion Regulation Scale (DERS): this is a 36-item self-evaluation scale of six subdomains of emotional regulation (awareness, clarity, goals, impulse, non-acceptance, strategies). Answers range from 1 “almost never” to 5 “almost always”, and higher total DERS scores reflect greater difficulties in regulating emotions [30].
- Reactivity, Intensity, Polarity and Stability questionnaire (RIPoSt-40): this scale was used to quantify emotional dysregulation in its different facets. It consists of 40 self-administered questions with answers given on a Likert scale going from 1 “never” to 6 “always”. Results can be divided into four subscales: affective instability, positive emotionality, negative emotionality and emotional impulsivity. A second-order scale can be identified as follows: negative emotion dysregulation, comprising affective instability, negative emotionality and emotional impulsivity [31].
- Hypomania Check List-32 (HCL-32): used to evaluate lifetime hypomanic symptoms, it consists of 32 questions with yes/no answers and has a two-factor structure (active/elated hypomania and risk-taking/irritable hypomania) [32].
- Brief Psychiatric Rating Scale (BPRS): 18-item tool commonly used by clinicians to assess current psychopathology and symptom severity [33].
- WHO Disability Assessment Schedule (WHODAS 2.0): a 36-item questionnaire used to explore self-reported functioning and disability in six major life domains: cognition, mobility, self-care, getting along, life activities, participation. Answers are given on a Likert scale ranging from 1 “no difficulty” to 5 “extremely difficult/cannot do” [34].

### 2.3. Data Analysis

Self-reported substance use was expressed by a four-level scale with increasing time of use: 0 = “never used” for non-SUD patients, 1 = “abstinent for more than one year”, implying use only in the past, 2 = “abstinent for less than one year” as recent use and 3 = “used for more than one year” as chronic use. We considered alcohol, THC and cocaine.

A principal component factor analysis was performed on clinical and symptomatological variables in order to reduce the heterogeneity among our A-ADHD patients. The factors which allowed the best possible reconstruction of the data were extracted by means of principal component analysis (PCA); we selected factors with an eigenvalue greater than 1. We then rotated our factor matrix according to Varimax with Kaiser normalization in order to achieve a simple structure and interpret the factor loadings. The loadings with a magnitude greater than the conventional cut-off of 0.40 were selected and used to describe the factors. In order to make factor scores comparable, they were standardized by converting them into z scores. All the subjects were then assigned to one of the different subtypes based on the highest z-scores obtained for each factor (predominant factor). This procedure gave us the opportunity to classify patients on the basis of the highest-ranking symptomatological cluster.

We also explored differences between the four different A-ADHD subtypes regarding demographic data, past psychiatric treatment and psychiatric comorbidities. For continuous variables, we used the Kruskal–Wallis test; for categorical variables, we used Pearson’s Chi-squared test with post hoc Bonferroni corrections.

Third, we performed a backward multinomial logistic regression to test clinical and demographic variables as possible predictive values of A-ADHD subtypes.

We used the statistical routines of IBM SPSS Statistics for Macintosh, Version 25.0 (Cupertino, CA, USA).

## 3. Results

### 3.1. Characteristics of the Sample

In our sample of 164 patients with a diagnosis of A-ADHD, 112 (68.3%) were males and 52 (31.7%) females. The mean age was 28.3 (sd = 10.7) years, mean body mass index was 24.3 (sd = 5.1). Of these 164 subjects, 42 (25.6%) were unemployed, and 122 (74.4%) were employed; the mean number of years of schooling was 13.40 (sd = 3.18). Regarding marital status, most of the subjects (140, 83.4%) were single, while 24 (16.6%) were married. A total of 85 (51.8%) subjects had received previous psychiatric drug treatment and more precisely, 53 (32.3%) with antidepressants, 37 (22.6%) with antipsychotics. Only 45 (27.4%) had been diagnosed which allowed them to receive specific treatments (methylphenidate or atomoxetine) for ADHD in childhood or adolescence.

Regarding psychiatric comorbidities, 134 (81.7%) of our patients had at least one comorbid psychiatric disorder: 66 (40.2%) had anxiety disorders, 66 (40.2%) had bipolar disorder, 7 (4.3%) had major depressive disorder, 72 (43.9%) had borderline personality disorder, 10 (6.1%) had obsessive compulsive disorder, 28 (17.1%) had specific learning disabilities, 13 (7.9%) had tic disorders, 8 (4.9%) had an intellectual disability, 13 (7.9%) had autism spectrum disorder, 19 (11.6%) had feeding and eating disorders and 60 (36.3%) had delayed sleep phase disorder.

### 3.2. Factor Analysis

Factor analysis, which is reported in Table 1, revealed a four-factor solution. Factor 1, Emotional Dysregulation (ED), included DERS-TOT score and RIPOST-ED score, which are the two rating scales for emotional dysregulation. TEMP-depressive, TEMP-cyclothymic and TEMP-anxious also loaded with factor 1, and they can be interpreted as indirect temperamental measures of emotional dysregulation. Lastly, the BARRAT-TOT score, ASRS-TOT score and WHODAS-TOT score measured impulsivity, self-reported ADHD symptoms and impairment in global functioning. Factor 2, Substance Use (SU), included the lifetime use of all substances (Alcohol, Cannabis, Cocaine-lifetime) and BPRS-TOT, which indicates a severe degree of general psychopathology. Factor 3, Core-ADHD Symptoms (Co-ADHD), included the three subscales of CAARS (inattentive, hyperactive-impulsive and the ADHD index). Factor 4, Positive Emotionality (PE), included TEMP-hyperthymic, RIPOST-positive emotionality and HSRS-Tot mean scores, all measures that give a direct or indirect assessment of positive emotionality. ED was the predominant factor in 45 patients, SU in 43, CO-ADHD in 37 and PE in 39.

### 3.3. Comparison of Demographic and Clinical Features Among Different Clinical Subtypes

The comparisons of demographic and clinical variables among the, predominantly, Emotional Dysregulation, Substance Use, Core-ADHD Symptoms and Positive Emotionality groups of patients are reported in Table 2. The four groups show similar results regarding age, body mass index, years of schooling, gender and marital status. A statistically significant difference was found in the employment rate, with more employed subjects in the PE-predominant group compared with the SU-predominant group of patients. Regarding past pharmacological psychiatric treatment, past antidepressant treatment was more strongly represented in the ED-predominant group compared with PE-predominant patients. The four groups did not show statistically significant differences for psychiatric comorbidity except with bipolar disorder, which was more strongly represented in SU-predominant patients than in CO-ADHD ones, and with borderline personality disorder, which proved to be more common in the ED-predominant group than in the CO-ADHD-predominant group (chi = 12.306, *p* = 0.006).

In the multinomial logistic regression analysis (Table 3), we studied the associations between the predominant ED, SU and PE groups, keeping as a reference point the predominantly CO-ADHD group.

A positive association was found for the ED-predominant group with borderline personality disorder. The SU-predominant group was positively associated with bipolar disorder and negatively with female gender, professionally employed and delayed sleep phase disorder. Lastly, the PE-predominant group was negatively associated with delayed sleep phase disorder.

## 4. Discussion

The demographic and clinical characteristics of our patients were consistent with those reported in other A-ADHD samples [35,36]. The main result of our study is the identification through factorial analysis of four clinical variants of A-ADHD, mainly distinguished by ED, SU, CAS and PE.

In a recent report, the DSM-5 distinction between predominantly inattentive, hyperactive/impulsive and combined subtypes [37] has been criticized, and two main clinical presentations have been proposed for A-ADHD: inattentive and emotionally dysregulated [38,39].

The importance of emotional dysregulation in the psychopathology of neurodevelopmental disorders has been confirmed by many empirical observations [17,40]. Most of the recent research has, however, been focused on the evaluation of the relationships between emotional dysregulation and DSM-5 core-ADHD features [39]. The traditional view does not consider other clinical implications that have been noted elsewhere [1].

Emotional dysregulation is characterized [41] by rapid mood swings, impulsive emotionality with related uncontrolled and maladaptive behavior and marked difficulties in regulating negative emotionality. The conceptualization of this emotional instability as a neurodevelopmental syndrome in itself postulates that it may represent a common neurophysiological substrate for several conditions described from different perspectives as neurodevelopmental, mood and personality disorders [42,43,44,45].

In our first factor, the severity of emotional dysregulation (measured by DERS-TOT and RIPOST-ED) and the associated affective temperaments (TEMP-depressive and TEMP-cyclothymic) are grouped together, so suggesting relative independence of the DSM-5 core-ADHD features. This result is consistent with other reports [39]. A significant level of impairment in overall functioning, as registered by WHODAS-TOT, is associated with the severity of emotional dysregulation. Many studies describe how co-occurring severe emotional dysregulation in neurodevelopmental disorders markedly worsens the basic functioning of the patient [25,39,46,47].

As for positive emotionality, in our sample, due to the presence of hyperthymic temperament, RIPOST-Positive Emotionality and HCL-32 total scores, it seems to be relatively independent of the severity of emotional instability, supporting the possible existence of an A-ADHD subtype with less difficulty in keeping emotional control [48,49] and a relatively high level of functioning.

Consistent with the view that substance use is very common in A-ADHD patients, we observed a high rate of SUD in our sample [50,51]. In our sample, Factors 2 and 3 identified clinical variants that are distinguished by lifetime substance use and by DSM-5 core-ADHD features as relatively independent. In other words, clinical variants for which the highest scores in measures of specific ADHD psychopathology have been reported are relatively independent of those with predominantly SUD features. This observation is partly in conflict with the literature, which shows a positive correlation between the severity of ADHD symptoms and those of SUD [52,53]. Some recent studies have, however, proposed that substance use is not associated with substantial changes in ADHD phenomenology [54]. Other reports have shown that substance use is more frequent in ADHD subtypes with severe emotional dysregulation and externalizing disorders, but we have not found any correlation with these specific features [55,56,57,58]. Factor 2 also includes the BPRS-TOT score for general psychopathology, which could indicate greater severity of global psychopathology in A-ADHD patients with SUD. This observation is true for most psychiatric disorders [59,60].

Comparative studies on demographic and clinical characteristics shown by the various clinical subtypes showed that patients with stable employment are more common in the PE than in the SU group. This observation is consistent with research showing that subjects with positive emotionality tend to be less sensitive to negative feedback while being better adapted socially [61]. They also avoid potentially damaging situations, such as the use of drugs [62]. Moreover, a cognitive focus on positive emotions may favor problem solving and decision making [63,64] and, consequently, the maintenance of a stable job.

We will now compare various previous treatments that rely on psychiatric drugs: as expected, ED-predominant patients were more frequently treated with antidepressants than those with predominant PE. Emotional dysregulation is, indeed, associated with negative affect and more frequently prompts medical attention. In addition, the prolonged use of antidepressants may be associated with emotional instability [65,66] and may increase the difficulty experienced in controlling stress and negative emotions in these patients [67,68,69].

Bipolar disorder and SUD frequently coexist [70,71] and this seems true even when bipolar disorder is associated with ADHD, as confirmed by our results. Lastly, as expected, in our sample borderline personality disorder is associated with ADHD variants, including severe emotional dysregulation. The association between these syndromic variants involves the reward systems that include brain structures such as the prefrontal cortex, the ventral striatum and the anterior cingulate [72,73]. Someone might object that almost half of the sample had borderline personality disorder, which suggests that this sample has more than just A-ADHD, demonstrating itself as a severely emotionally dysregulated sample. This level of A-ADHD comorbidity with BPD is extremely and unusually high, suggesting this sample is not generalizable to a standard population of A-ADHD. Reimherr’s work underlines this aspect: adults with ADHD may receive suboptimal interventions because of the misconception that they reflect a different diagnosis, such as a personality disorder; this potential for misdiagnosis has been identified by others [39,74].

Gender differences resulted when multinomial logistic regression was applied, with A-ADHD females showing a lower risk of having SUD. The same finding was reported by Sobanski et al. [75], which highlights the fact that ADHD females are more likely to develop comorbidities with depressive episodes and eating disorders, while males are more frequently affected by SUD.

It is known that females have greater difficulty in regulating negative emotions in respect to males; this leads to a propensity towards comorbidities in the emotional spectrum and anxiety disorders [76,77,78], while men usually have greater impulsiveness, which leads more easily to addiction [79,80]. Delayed circadian rhythm disorder was a risk factor for inclusion in the CAS group, suggesting a strong relationship between circadian rhythm disturbances and neurodevelopmental disorders [81,82,83]. It is known that delayed circadian rhythm and ADHD have a common genetic basis and possible shared pathophysiology [84].

Limitations: In our sample, there was an imbalance between males and females, which may skew the generalization of our results. Additionally, the sample was relatively uneducated, lacking college graduates and also lacked marital statuses other than single, which is not generalizable to most of the adult population. However, our sample gives a good description of individuals with A-ADHD who come for a consultation at a level II psychiatric clinic. As an additional issue, most of our A-ADHD subjects had never been diagnosed with the disorder, they had never been medicated or were aware of their symptoms. Thus, they may not have had the awareness to report accurately on their ADHD symptoms. However, this fact often happens in real life; narrative inconsistency and alexithymia are typical of patients with A-ADHD. Thus, the clinical variants highlighted by us are not negatively affected by that. At the most, this fact accounts for the diagnostic difficulties encountered by psychiatrists.

## 5. Conclusions

In this study, we have described four clinical variants of A-ADHD, distinguished as ED, SU, CAS and PE. Predominantly ED patients showed worse overall functioning, frequent early treatment with antidepressants and common association with borderline personality disorder. A-ADHD patients with predominant SU were frequently males with comorbid bipolar disorder who showed severe general psychopathology. The predominantly CAS variant showed co-occurring delayed sleep phase, and the PE variant was distinguished by hyperthymic temperament and hypomania, with a relatively high level of functioning.

Our empirically based description of four clinical variants of A-ADHD shows several aspects that elaborate on details of the DSM-5 diagnostic criteria. This clinical perspective may have pertinent implications for patient identification and management in various psychiatric settings.

## Figures and Tables

**Table 1:** Factor structure of Emotional Dysregulation, Substance Use, Core-ADHD Symptoms and Positive Emotionality dimensions. *N* = 164.

Variables	Emotional Dysregulation	Substance Use	Core-ADHD Symptoms	Positive Emotionality
Cannabis use		0.77		
ALCOHOL use		0.71		
COCAINE use		0.80		
CAARS-inattentive			0.83	
CAARS-hyperactive			0.70	
CAARS-index			0.87	
ASRS-TOT	0.64			
TEMP-depressive	0.73			
TEMP-cyclothymic	0.79			
TEMP-hyperthymic				0.73
TEMP-irritable	0.43	0.44		
TEMP-anxious	0.65			
BARRAT-TOT	0.51	0.44		
DERS-TOT	0.72			
RIPOST-ED	0.82			
RIPOST-positive emotionality				0.70
HCL-32-TOT				0.59
BPRS-TOT		0.44		
WHODAS-TOT	0.62			
Variance (%)	28.54	12.70	9.37	7.90
Prominent dimension *N* (%)	45 (27.4)	43 (26.2)	37 (22.5)	39 (23.7)

Extraction method: principal component analysis. Rotation Method: Varimax with Kaiser Normalization. ASRS-TOT = Adult ADHD Self-Report Scale total score; TEMPS = Temperament Evaluation of Memphis, Pisa, Paris and San Diego; BARRAT-TOT = Barratt Impulsiveness Scale total score; DERS-TOT = Difficulties in Emotion Regulation Scale total score; RIPOST = Reactivity Intensity Polarity Stability Questionnaire; HCL-32 = Hypomania Checklist total score; BPRS-TOT = Brief Psychiatric rating scale total score; WHODAS = World Health Organization Disability Assessment Schedule.

**Table 2:** Demographic and clinical characteristics, comparisons between dominant subgroups.

Tools Score	Emotional Dysregulation *N* = 45	Substance Use *N* = 43	Core-ADHD Symptoms *N* = 37	Positive Emotionality *N* = 39	Statistics	
	M ± sd	M ± sd	M ± sd	M ± sd	F	*p*
Age	26 ± 15	24 ± 7	23 ± 10	25 ± 26	4.92	0.178
Body Mass Index	23.60 ± 3.0	24.85 ± 5.6	22.40 ± 4.7	23.60 ± 3.8	1.26	0.738
Years of schooling	13 ± 4	13 ± 4	13 ± 2.5	13 ± 1	5.40	0.144
	N (%)	N (%)	N (%)	N (%)	Chi^2^	*p*
Female gender	20 (44.4) a	8 (18.6) a	11 (29.7) a	13 (33.3) a	6.89	0.075
Professionally employed	34 (75.6) ab	25 (58.1) b	28 (75.7) ab	35 (89.7) a	10.85	0.013
Married	8 (17.8) a	2 (4.7) a	7 (18.9) a	7 (17.9) a	4.67	0.197
Past psychiatric treatment	28 (62.2) a	23 (53.5) a	17 (45.9) a	17 (43.6) a	3.56	0.312
Past antidepressant treatment	22 (48.9) a	14 (32.6) ab	10 (27.0) ab	7 (17.9) b	9.80	0.020
Past antipsychotic treatment	8 (17.8) a	15 (34.9) a	5 (13.5) a	9 (23.1) a	6.06	0.108
Adolescent ADHD treatment	12 (26.7) a	12 (27.9) a	11 (29.7) a	10 (25.6) a	0.17	0.981
Psychiatric comorbidity						
Anxiety disorders	23 (51.1) a	16 (37.2) a	15 (40.5) a	12 (30.8) a	3.83	0.280
Bipolar disorder	22 (48.9) ab	23 (53.5) b	8 (21.6) a	13 (33.3) ab	10.64	0.014
Major depressive disorder	3 (6.7) a	1 (2.3) a	2 (5.4) a	1 (2.6) a	1.42	0.700
Obsessive compulsive disorder	4 (8.9) a	2 (4.7) a	2 (5.4) a	2 (5.1) a	0.86	0.834
Feeding and eating dis.	6 (13.3) a	6 (14.0) a	3 (8.1) a	4 (10.3) a	0.87	0.832
Intellectual disability	1 (2.2) a	3 (7.0) a	2 (5.4) a	2 (5.1) a	1.12	0.772
Autism spectrum dis.	6 (13.3) a	2 (4.7) a	4 (10.8) a	1 (2.6) a	4.39	0.222
Specific learning disability	7 (15.6) a	9 (20.9) a	7 (18.9) a	5 (12.8) a	1.11	0.774
Tic disorder	2 (4.4) a	7 (16.3) a	2 (5.4) a	2 (5.1) a	5.59	0.133
Borderline personality disorder	28 (62.2) a	21 (48.8) ab	11 (29.7) b	12 (30.8) b	12.30	0.006
Delayed sleep phase	19 (42.2) a	15 (34.9) a	18 (48.6) a	8 (20.5) a	7.33	0.062

Letters denotes a subset of factor categories whose column proportions are not very different from each other (0.05).

**Table 3:** Multinomial logistic regression of demographic and clinical characteristics compared with the factorial structure: only significant values are reported.

Parameter Estimates ^1^	B	OR	95% CI	*p*
ED patients (*N* = 45)				
Borderline personality disorder	1.32	3.76	(1.29–10.89)	0.010
SU patients (*N* = 43)				
Female gender	−1.47	0.23	(0.06–0.76)	0.017
Professionally employed	−1.08	0.33	(0.11–0.98)	0.047
Bipolar disorder	1.31	3.73	(1.28–10.86)	0.016
Delayed sleep phase	−1.06	0.34	(0.12–0.098)	0.047
PE patients (*N* = 39)				
Delayed sleep phase	−1.32	0.27	(0.08–0.80)	0.019

^1^ Predominantly Co-ADHD group (*N* = 37) as the reference category; ED = Emotionally Dysregulation; SU = Substance Use; Co-ADHD = Core-ADHD symptoms; PE = Positive Emotionality.

## Data Availability

The data are not publicly available due to the privacy reasons.
